# Diagnostic Performance of CXR and CT in Pediatric Foreign Body Aspiration: A PICU-Based Bronchoscopy Study

**DOI:** 10.3390/children12081035

**Published:** 2025-08-07

**Authors:** Mustafa Orhan Duyar, Mehmet Akif Dündar, Sinem Nisa Karadeli, Murat Doğan

**Affiliations:** 1 Department of Pediatrics, Kayseri City Training and Research Hospital, Health Sciences University, Kayseri 38039, Turkey; sinemnisa.karadeli@saglik.gov.tr; 2 Division of Pediatric Intensive Care, Department of Pediatrics, Kayseri City Training and Research Hospital, Health Sciences University, Kayseri 38039, Turkey; mehmetakifdundar@erciyes.edu.tr; 3 Division of Pediatric Emergency, Department of Pediatrics, Kayseri City Training and Research Hospital, Health Sciences University, Kayseri 38039, Turkey; murat.dogan33@saglik.gov.tr

**Keywords:** pediatric intensive care, foreign body aspiration, bronchoscopy, chest radiography, computed tomography, organic aspiration, airway obstruction

## Abstract

Objective: To evaluate the clinical, radiological, and bronchoscopic features of pediatric patients admitted to the pediatric intensive care unit (PICU) with suspected foreign body aspiration (FBA), and to compare the diagnostic performance of chest radiography (CXR) and computed tomography (CT). Methods: We retrospectively analyzed 71 children admitted to the PICU of Kayseri City Training and Research Hospital for suspected tracheobronchial FBA between January 2020 and December 2024. Demographic data, clinical presentations, imaging findings, bronchoscopic results, and outcomes were recorded. The sensitivity, specificity, positive predictive value (PPV), and negative predictive value (NPV) of CXR and CT were calculated using bronchoscopy as the reference standard. Results: The mean age was 2.61 ± 3.59 years, and 66.1% were male. Organic materials were the most commonly aspirated objects, especially in children aged 0–3 years. The right main bronchus was the most frequently affected site. CXR had a sensitivity of 94.9% (95% CI: 83.1–98.6) and a specificity of 71.0% (95% CI: 53.4–83.9), while CT had a sensitivity of 63.2% (95% CI: 41.0–80.9) and a specificity of 100% (95% CI: 87.5–100.0). Bronchoscopy revealed no foreign body in 45.1% of cases. Most patients (94.4%) fully recovered; complications included two deaths, one lobectomy, and one case of hypoxic sequelae. Conclusion: FBA remains a critical pediatric emergency, particularly in young children. CXR is a highly sensitive and accessible screening tool, while CT offers high specificity but lower sensitivity. Prompt diagnosis and bronchoscopy by experienced teams ensure favorable outcomes.

## 1. Introduction

Tracheobronchial foreign body aspiration (FBA) is a pediatric emergency that requires prompt evaluation. Although it can occur at any age, it is most frequently observed in children between 6 months and 3 years of age [1,2,3]. Foreign body aspiration is a significant cause of mortality in children under the age of three and represents a serious public health concern. In this age group, it ranks as the fourth most common cause of accidental death and accounts for approximately 7% of all deaths in children under the age of four. This highlights the need to increase awareness and to develop effective preventive strategies targeting early childhood [4,5,6,7]. Due to their immature airway reflexes, anatomical predisposition, inability to distinguish edible from inedible objects, and evolving feeding skills, children under the age of three are at the highest risk of aspiration [8,9,10].

Patients often present with a history of suspected aspiration, accompanied by non-specific physical and radiological findings that range from normal to pathological. The clinical presentation may vary widely in severity, from mild respiratory distress to respiratory arrest [1,11,12,13]. The severity of symptoms depends on the type of aspirated material, the age of the child, the time elapsed before medical intervention, the anatomical location of the obstruction, and the degree of airway blockage [6,9]. Silent or non-specific symptoms may delay diagnosis and increase the risk of complications. Therefore, there is a critical need for reliable clinical and radiological indicators that can expedite the diagnostic process and guide appropriate intervention.

Although the literature contains numerous studies on pediatric FBA, there is a paucity of data that comprehensively assess the pre- and post-bronchoscopy clinical course, diagnostic value of radiological imaging, characteristics of the aspirated materials, and complication rates in an integrated manner. Moreover, data derived from patients followed in pediatric intensive care units (PICUs) offer critical insight into the severe clinical spectrum of FBA [14,15,16].

The aim of this study is to analyze the demographic, clinical, and radiological characteristics of patients who were admitted to the PICU with suspected FBA and underwent bronchoscopy, to identify key diagnostic indicators, and to compare the diagnostic performance of posteroanterior chest radiography (CXR) and contrast-enhanced computed tomography (CT) in order to obtain practical implications for clinical decision-making.

## 2. Materials and Methods

This retrospective study included pediatric patients who were admitted either preoperatively or postoperatively to the pediatric intensive care unit (PICU) of Kayseri City Training and Research Hospital, Turkey, between 1 January 2020 and 31 December 2024 for bronchoscopy due to suspected tracheobronchial foreign body aspiration (FBA). Patients who were recommended to undergo bronchoscopic evaluation at the time of admission to the emergency department but did not provide consent for the procedure were not admitted to the PICU and were therefore excluded from the study. During our study, there were no patients who refused the procedure after ICU admission. Except for one patient, all patients admitted to the ICU underwent a posteroanterior chest radiograph (CXR) in the emergency department where they initially presented, and most also underwent contrast-enhanced thoracic CT according to the medical approach of the thoracic surgery specialists consulted prior to the procedure. In this context, no additional factors that could affect imaging preferences were considered, and thus the distribution of imaging modalities was assumed to be homogeneous across the patient cohort.

In the study, variables such as patient age, type of aspirated material, anatomical region from which the material was removed, time to hospital admission, the day bronchoscopy was performed based on the day of admission, whether FBA was mentioned or suspected in family history, presenting complaints, physical examination and radiological findings, preoperative and postoperative clinical status, length of hospital stay, need for oxygen support or mechanical ventilation, and discharge or exitus status were retrospectively obtained by reviewing the hospital automation system and patient files and recorded into the dataset.

Pediatric patients presenting with suspected FBA were initially evaluated in the pediatric emergency department. Patients for whom bronchoscopy was planned were generally admitted to the pediatric intensive care unit (PICU) for pre-procedural stabilization and subsequently transferred to the operating room. In cases where no PICU bed was available, some patients were temporarily monitored in pediatric wards until the procedure. All patients included in the study were monitored postoperatively in the PICU.

Radiologic evaluations included preoperative posteroanterior CXR and contrast-enhanced thoracic CT. When evaluating the diagnostic test performance, true positive (TP), false negative (FN), false positive (FP), and true negative (TN) values were used. CXR findings including unilateral increased aeration, thickened pulmonary fissure, atelectasis, or significant infiltrates were considered significant. CT findings were considered significant if the radiology report clearly indicated suspicion of FBA. These radiologic findings were compared with bronchoscopy results to assess the diagnostic performance of each imaging modality.

This study was conducted in accordance with the principles of the Declaration of Helsinki and approved by the Institutional Ethics Committee of Health Sciences University, Kayseri City Training and Research Hospital (Approval No: 340, Date: 25 February 2025).

### Statistical Analysis

Data were analyzed using IBM SPSS Statistics for Windows, Version 25.0 (IBM Corp., Armonk, NY, USA). Categorical variables were expressed as frequencies (*n*) and percentages (%), while continuous variables were presented as mean ± standard deviation or median (minimum–maximum), depending on the distribution. To evaluate the diagnostic performance of imaging modalities, bronchoscopy was accepted as the reference standard, and sensitivity, specificity, positive predictive value (PPV), and negative predictive value (NPV) were calculated for chest X-ray and computed tomography. No comparative statistical tests were applied in this study. A *p*-value of <0.05 was considered statistically significant.

## 3. Results

During the study period, bronchoscopy was performed on 71 patients with suspected tracheobronchial foreign body aspiration (FBA). Of the patients, 66.1% were male (*n* = 47) and 33.8% were female (*n* = 24). The mean age was 2.61 ± 3.59 years. The majority of patients presenting to the pediatric emergency department (*n* = 71, 100%) were monitored in the pediatric intensive care unit (PICU) (*n* = 57, 80.28%) during the preoperative period, while a smaller portion were admitted to the ward (*n* = 10, 14.08%). Four patients (5.63%) were referred directly from the emergency department (*n* = 4, 5.63%) to surgery. All patients (*n* = 71, 100%) were monitored in the PICU postoperatively. The most common presenting symptoms were cyanosis and sudden onset of coughing (*n* = 39, 54.9%), followed by dyspnea and wheezing (*n* = 22, 31.0%), asymptomatic aspiration (*n* = 4, 5.6%), vomiting (*n* = 3, 4.2%), chronic cough (*n* = 1, 1.4%), and rare presentations such as cardiac arrest and syncope (one case each, 1.4%). The vast majority of cases (94.20%, *n* = 65) presented to the hospital with direct witnessing or suspicion of FBA, with 94.36% (*n* = 67) mentioning suspicion of foreign body aspiration in the initial patient history and 95.77% (*n* = 68) mentioning it upon detailed questioning. In the preoperative period, 87.3% (*n* = 62) of patients were followed with room air or nasal oxygen support, 8.5% (*n* = 6) were intubated, and 4.2% (*n* = 3) received high-flow nasal cannula (HFNC) support. Postoperatively, 84.5% (*n* = 60) were transitioned to room air or nasal oxygen, 12.7% (*n* = 9) remained intubated, and 2.8% (*n* = 2) continued with HFNC. The majority of patients (94.4%, *n* = 67) were discharged with full recovery. Two patients (2.8%) died due to bronchospasm and respiratory failure, one patient (1.4%) underwent lobectomy, and one (1.4%) developed hypoxic sequelae due to complete obstruction. Detailed demographic and clinical data are shown in Table 1.

The median hospital stay was 2 ± 2.98 days (range: 1–18), with a median admission delay of 1 ± 3.17 days (range: 1–23). Bronchoscopy was typically performed on the first day of hospitalization (median 1.0 ± 1.19 days; range: 1–10). Post-bronchoscopy, the median length of hospital stay was 1 ± 3.0 days (range: 0–15). Preoperative HFNC support lasted a median of 1 ± 5.19 days, while intubated patients received mechanical ventilation for 1 ± 0.38 days. Postoperatively, HFNC was continued for 1 day in two patients, and mechanical ventilation was continued for a median of 2 ± 5.45 days in intubated patients. Details regarding hospital stay and respiratory support are summarized in Table 2.

In 45.07% (*n* = 32) of the patients who underwent bronchoscopy, no foreign body was detected, while in 25.32% (*n* = 18), ground organic matter (indistinguishable material detected during bronchoscopy upon suspicion of aspiration of formula, nuts, boiled corn, beans, and apricots as indicated by families) was identified. The most frequently affected site was the right main bronchus (43.7%, *n* = 31), followed by the right lower lobe (19.7%, *n* = 14) and the left lower lobe (16.9%, *n* = 12). In patients aged 0–3 years, organic matter aspiration was seen in 96.8% (*n* = 30) of cases. All corn aspirations and 75% of sunflower seed aspirations occurred in this age group. The most common anatomical site in this age group was the right main bronchus (41.9%, *n* = 13), followed by the left main bronchus (32.3%, *n* = 10) (Table 3).

Among patients followed on room air preoperatively, 4.8% (*n* = 3) required intubation postoperatively, and one patient (1.1%) required HFNC. Of the three patients supported with HFNC preoperatively, two were transitioned to room air and one continued with HFNC after the procedure (Table 4).

Radiologic evaluations showed that 98.6% (*n* = 70) of patients underwent preoperative CXR, and 64.8% (*n* = 46) underwent contrast-enhanced thoracic CT. Based on bronchoscopy results as reference, the sensitivity, specificity, positive predictive value (PPV), and negative predictive value (NPV) of CXR were calculated as 94.9%, 71.0%, 80.4%, and 91.7%, respectively (TP = 37, FN = 2, FP = 9, TN = 22). The sensitivity, specificity, PPV, and NPV of CT were found to be 63.2%, 100%, 100%, and 79.4%, respectively (TP = 12, FN = 7, FP = 0, TN = 27). In the subgroup with only ground organic matter aspiration, CXR was positive in all patients (18/18) and its sensitivity was determined as 100%; however, since there were no negative CXR results in this group, the specificity, PPV, and NPV could not be calculated. In the subgroup with ground organic matter aspiration, CXR demonstrated 100% sensitivity, but the specificity and predictive values could not be calculated due to the absence of negative results. CT sensitivity in this subgroup was 50%, with the predictive values similarly not calculable (Table 5).

## 4. Discussion

The primary aim of this study was to evaluate the clinical features of children admitted to the pediatric intensive care unit (PICU) with suspected foreign body aspiration (FBA), before and after bronchoscopy, to identify associated risk factors, and to compare the diagnostic performance of radiological imaging modalities. Our findings revealed that being aged 0–3 years and male sex were significant risk factors for FBA. Organic material aspiration was prevalent across all age groups, with granular foods such as corn and sunflower seeds particularly common in younger children. The right main bronchus was the most frequently involved anatomical site. The sensitivity of chest X-rays and the PPV and specificity of CT were extremely high. A positive family history was found to be highly reliable, and both radiographic findings and clinical symptoms played a crucial role in diagnosis. This study reaffirms that FBA is a significant pediatric emergency with potentially serious consequences across all age groups. In our cohort, 76% of patients were aged 0–3 years, and 66.1% were male, consistent with the previous literature reporting [17,18] that 75.5% of FBA cases occur in children under three and 61.2% in males.

The high positivity rate (94.36%) of FBA suspicion in family history and the consistency of presenting symptoms (sudden cough, cyanosis, wheezing) with the literature [2,13,17,18,19,20] once again support the role of patient history in the diagnostic process [17,18,20,21,22,23]. Although rare, cases presenting with syncope or cardiopulmonary arrest should prompt the consideration of FBA as a differential diagnosis [24,25]. In our study, such presentations were infrequent (1.4% each), but underscore the importance of maintaining a low threshold for clinical suspicion.

Although some cases of FBA may be asymptomatic [1,8,26], detailed data on pre- and postoperative respiratory support needs are limited in the literature. In this study, 87.3% of patients required no supplemental oxygen preoperatively, while 8.5% were intubated. Postoperatively, only 4.8% of patients previously on room air required intubation. Among patients receiving preoperative HFNC support, 66.7% were transitioned to room air after bronchoscopy. These findings support the safety and effectiveness of bronchoscopy when performed by experienced teams, with rapid clinical improvement observed in most cases.

Most of the aspirated materials were organic in nature (89.7%), with this rate increasing to 96.7% in children aged 0–3 years. These results align with existing studies, which highlight the predominance of organic substances such as nuts and seeds [1,12,18,26,27,28]. Corn, sunflower seeds, and walnuts were the most frequently aspirated items, consistent with prior research [29]. Recent studies have shown that the proportion of patients in whom material was not detected during bronchoscopy decreased from 37% to 17% with the CT usage protocol in FBA cases, whereas this rate was around 39.1% in bronchoscopies performed without imaging [23,30]. However, some studies have shown that the rate of negative bronchoscopy still varies widely, ranging from 7% to as high as 67% [2,12]. Although our negative bronchoscopy rate (45.1%) is above the literature average, performing the procedure in patients with high clinical suspicion despite no pathological findings on imaging and the predominance of the 0–3 age group in our study explains this situation. In the literature, it is emphasized that negative bronchoscopies are inevitable in this patient group and delayed procedure increases the risk of complications [31,32].

Regarding anatomical localization, the right main bronchus was most frequently affected (43.7%), particularly among children aged 0–3 years (41.9%). This distribution is consistent with known anatomical angulation differences between the right and left bronchi [29,33]. In young children, the right main bronchus is anatomically shorter, wider, and more vertically aligned compared to the left, which predisposes it to foreign body entry. These structural characteristics make the right bronchial tree the path of least resistance for aspirated materials, especially in early childhood when protective airway reflexes are also less mature [9,10].

Data regarding the reliability of radiological findings are varied in the literature. Extensive literature reviews indicate that only 11% of foreign bodies have radiopaque properties and therefore chest radiographs may appear normal [1,29]. One study reported that CXR was normal in 47.1% of FBA cases [14], while another study stated that pathological findings were detected in 27.1% of children with suspected FBA on standard chest X-ray and in 52.9% on expiratory-phase chest X-ray [1]. Several studies have reported that the sensitivity of chest X-ray after FBA ranged between 27 and 88% and specificity between 30 and 77%, with conflicting rates [22,28,34,35]. In a study by Moreau et al., the overall sensitivity, specificity, PPV, and NPV for expiratory-phase chest X-ray in suspected FBA were determined as 94.4%, 91.1%, 91.9%, and 93.9%, respectively [1]. Recent studies have highlighted the high sensitivity (91–100%) and specificity (98–100%) of CT (6,8,14). Gibbons et al. emphasized that in bronchoscopy procedures coordinated with CT, there was a 94.4% positive material detection rate, thus preventing negative bronchoscopies [23]. Ahmed O. et al. found the PPV of low-dose non-contrast airway CT in detecting FBA to be 100% and the NPV to be 97% [30]. In our study, the diagnostic performances of CXR and CT in the diagnosis of FBA in children were also compared. Our findings revealed that the high sensitivity of CXR (94.9%) makes it an important screening tool for FBA. This increases the value of CXR, especially as it is easily accessible, low cost, and rapid. In our study, the specificity and PPV of CT were found to be 100%. This indicates that positive findings detected by CT were largely confirmed by bronchoscopy and supports the literature. However, in our study, the sensitivity of CT was lower than in indices (63.2%), suggesting that false negative results may occur in some cases. This can be explained by the high rate (*n* = 18, 25.32%) of ground organic matter aspiration observed in our study. Our study shows that CXR is more sensitive than CT (CXR = 100%, CT = 50%) in ground organic foreign body aspirations. Furthermore, the disadvantages of CT such as radiation exposure, cost, and the need for sedation should be considered [1,35,36]. In the presence of clinical suspicion, negative radiological imaging should not rule out the diagnosis, and the indication for bronchoscopy should persist.

FBA-related complications can occur both at the time of aspiration and during bronchoscopy [13,29]. Major complication rates up to 13.7% have been reported, with the highest incidence seen in children aged 1–3 years. Mortality rates remain relatively low at approximately 0.3% [13,26]. In our study, mortality occurred in two patients (2.8%) due to bronchospasm and respiratory failure. One patient (1.4%) required lobectomy, and one developed hypoxic sequelae following cardiac arrest. A total of 94.4% of patients were discharged with full recovery. These outcomes highlight the importance of early diagnosis and management by experienced teams in improving prognosis.

This study is limited by its retrospective design and single-center data collection. Additionally, since it was conducted exclusively on patients who consented to bronchoscopy and were subsequently monitored in the pediatric intensive care unit, it does not include short- or long-term data on patients with suspected foreign body aspiration in the emergency department who declined the procedure. Nevertheless, our findings provide valuable insights into the management and diagnosis of pediatric foreign body aspiration in critical care settings.

## 5. Conclusions

This study demonstrates that FBA in childhood predominantly affects male children aged 0–3 years, with granular organic foods being the most common causative agents. A detailed and positive family history, along with characteristic symptoms such as sudden cough and cyanosis, plays a critical role in prompting bronchoscopy, especially in high-risk age groups.

Among diagnostic tools, posteroanterior CXR showed high sensitivity, including for radiolucent organic materials, making it a valuable and accessible screening method. CT, while highly specific, showed limited sensitivity and should not be solely relied upon to exclude FBA in the presence of clinical suspicion. Bronchoscopy remains the gold standard for diagnosis and treatment and can be performed safely in experienced centers with a high rate of clinical recovery and a low complication profile.

Given the potentially fatal consequences of FBA in young children, especially those aged 0–3 years, public education, early clinical recognition, and prompt intervention are essential components of effective management.

## Figures and Tables

**Table 1:** Demographic and clinical characteristics of the patients.

Variables	N	%
Unit (Pre-op period)		
PICU	57	80.2
Pediatric ward	10	14.0
Emergency	4	5.6
Unit (Post-op period)		
PICU	71	100.0
Gender		
Male	47	66.1
Female	24	33.8
Reason for Presentation		
Bruising and sudden coughing	39	54.9
Shortness of breath and wheezing	22	30.9
FBA without symptoms	4	5.6
Vomiting	3	4.2
Chronic cough	1	1.4
Arrest	1	1.4
Syncope	1	1.4
Hospitalization Diagnosis		
FBA suspicion	65	94.2
Acute bronchiolitis	3	4.3
Lobar pneumonia	1	1.4
Patient History of FBA		
Reported	67	94.3
Not reported	4	5.6
FBA History Upon Questioning		
Suspected by family	68	95.7
Not suspected by family	2	2.8
Indeterminate	1	1.4
Pre-op Respiratory Support		
Room air	62	87.3
Mechanical ventilator	6	8.4
HFNC	3	4.2
Post-op Respiratory Support		
Room air	60	84.5
Mechanical ventilator	9	12.6
HFNC	2	2.8
Discharge Information		
Discharged	67	94.3
Deceased	2	2.8
Lobectomy	1	1.4
Hypoxic sequelae	1	1.4

PICU: pediatric intensive care unit, FBA: foreign body aspiration, HFNC: high-flow nasal cannula.

**Table 2:** Data on patient hospitalizations and respiratory support needs.

Variable	N	Average	SD	Median	%25	%50	%75	Min	Max
Age	71	2.6	3.5	1	1	1	3	0	15
Number of hospitalization days	71	3.0	2.9	2	2	2	3	1	18
What is the day of the complaint?	71	1.8	3.1	1	1	1	1	1	23
Day of procedure (after hospitalization)	71	1.1	1.1	1	1	1	1	1	10
Pre-op HFNC days	3	4.0	5.1	1	1	1	10	1	10
Post-op HFNC days	2	1.0	0.0	1	1	1	1	1	1
Pre-op MV days	6	1.1	0.3	1	1	1	1	1	2
Post-op MV days	9	5.0	5.4	2	1	2	9.5	1	15
Number of days pre-op	71	0.2	1.3	0	0	0	0	0	10
Number of days post-op	71	2.1	3.0	1	1	1	2	0	15

SD: standard deviation, MV: mechanical ventilator, HFNC: high-flow nasal cannula, Pre-op: preoperative, Post-op: postoperative.

**Table 3:** Detailed data on bronchoscopy results.

**Bronchoscopy result**	**N**	**%**
No material seen	32	45.0
Ground organic matter	18	25.3
Sweetcorn	5	7.0
Core	4	5.6
Peanut	1	1.4
Walnut	1	1.4
Formula	2	2.8
Green almond	1	1.4
Wooden toy part	1	1.4
Food	2	2.8
Lead	1	1.4
Nail	1	1.4
Pin	1	1.4
Package wire	1	1.4
**Place of origin of the material**	**N**	**%**
Right main bronchus	31	43.0
Lower right lobe	14	19.7
Lower left lobe	12	16.9
Trachea	4	5.6
Left main bronchus	3	4.2
Right upper lobe	3	4.2
Left upper lobe	1	1.4
Hypopharynx	1	1.4
Right and left main bronchi	1	1.4
**Bronchoscopy result in 0–3 age group**	**N**	**%**
Organic material	30	96.7
İnorganic material	1	3.2
Right main bronchus	13	41.9
Left main bronchus	10	32.2

**Table 4:** Patients’ oxygen support needs.

Pre-op O_2_ Support	Post-op O_2_ Support	N	%
Room air (*n*:62)	Room air	58	93.5
	HFNC	1	1.6
	Mechanical ventilator	3	4.8
HFNC (*n*:3)	Room air	2	66.6
	HFNC	1	33.3
Mechanical ventilator (*n*:6)	Mechanical ventilator	6	100.0

HFNC: high-flow nasal cannula, O_2_: oxygen, Pre-op: preoperative, Post-op: postoperative, MV: mechanical ventilator.

**Table 5:** Predictive data of radiologic imaging for FBA.

	Sensitivity (%, CI)	Specificity (%, CI)	PPV (%, CI)	NPV (%, CI)
All patients examined				
CXR (*n* = 70)	94.9 (83.1–98.6)	71.0 (53.4–83.9)	80.4 (66.8–89.3)	91.7 (74.2–97.7)
CT (*n* = 46)	63.2 (41.0–80.9)	100.0 (87.5–100.0)	100.0 (75.8–100.0)	79.4 (63.2–89.7)
Bronchoscopy revealed ground organic matter				
CXR (*n* = 18)	100.0 (82.4–100.0)	-		
CT (*n* = 10)	50.0 (23.7–76.3)	-		

CT: contrast-enhanced thorax computer tomography, CXR: chest radiography, FBA: foreign body aspiration, PPV: positive predictive value, NPV: negative predictive value, CI: 95% confidence interval, *n*: refers to the number of patients in each subgroup who had the specified radiologic evaluation.

## Data Availability

The data presented in this study are available on request from the corresponding author. The data are not publicly available due to patient privacy restrictions.
